# Neutrophil Plasticity and NETosis in Tumour Microenvironment: Tumour Evolution and Therapy Resistance

**DOI:** 10.1155/jimr/5568021

**Published:** 2026-01-06

**Authors:** Mojdeh Soltani, Sara Falahi, Mohammad Abbaszadeh, Mark J. M. Sullman, Hamed Fouladseresht, Nahid Eskandari

**Affiliations:** ^1^ Department of Immunology, Isfahan University of Medical Sciences, Isfahan, Iran, mui.ac.ir; ^2^ Department of Social Sciences, University of Nicosia, Nicosia, Cyprus, unic.ac.cy; ^3^ Department of Life and Health Sciences, University of Nicosia, Nicosia, Cyprus, unic.ac.cy; ^4^ Department of Immunology, Applied Physiology Research Center, Cardiovascular Research Institute, Isfahan University of Medical Sciences, Isfahan, Iran, mui.ac.ir

**Keywords:** immunotherapy, NETosis, neutrophil, tumour microenvironment

## Abstract

Neutrophil extracellular traps (NETs) are web‐like formations consisting of DNA‐histone complexes and associated proteins released from activated neutrophils. While NET formation plays an important role in innate immunity, it is also associated with the pathogenesis of autoimmune disorders such as rheumatoid arthritis, psoriasis and systemic lupus erythematosus. Research suggests that NETosis (the process of NET formation) may contribute to the progression of cancer and the spread of malignant tumours. A clear link exists between the accumulation of neutrophils in the tumour microenvironment (TME), known as tumour‐associated neutrophils (TANs) and NETosis activation in both primary and metastatic tumours. Furthermore, the literature highlights the role of NETs in modulating immune surveillance within the TME. This review aims to analyse the interplay between NETosis and the TME, emphasising its implications for tumour progression, immune evasion and resistance to therapy.

## 1. Introduction

The tumour microenvironment (TME) is a heterogeneous ecosystem primarily consisting of tumour cells and stromal cells, such as vascular endothelial cells and cancer‐associated fibroblasts (CAFs). Additionally, immune cells are crucial components of the TME, playing a significant role in either inhibiting or promoting cancer progression. Immune cells that inhibit tumour growth include effector T cells (such as CD4+ helper T cells and CD8+ cytotoxic T cells), natural killer (NK) cells, M1‐polarised macrophages, dendritic cells (DC) and N1‐polarised neutrophils. Conversely, immune cells that promote tumour growth include M2‐polarised macrophages, regulatory T cells (Tregs), myeloid‐derived suppressor cells (MDSCs) and N2‐polarised neutrophils.

Innate immune cells shape the TME by secreting various mediators, such as cytokines, metabolites, chemokines and other factors. Histopathological analyses of the immune components within the TME—encompassing both innate and adaptive immune cells—are crucial for cancer diagnosis, staging and evaluating prognosis or treatment response.

Neutrophils, also referred to as polymorphonuclear leukocytes (PMNs), arise from the myeloid lineage and are the most common circulating white blood cells in mice and humans. They serve as the first responders during inflammation, infection and injury. Neutrophils capture invading microorganisms and pathogens through processes such as degranulation, phagocytosis and the formation of neutrophil extracellular traps (NETs). While they play an important role in responding to acute infections and tissue injuries, they are also significantly involved in chronic inflammatory conditions, including cancer.

The role of neutrophils, specifically tumour‐associated neutrophils (TANs), within the TME remains a subject of considerable debate. TANs interact with other immune cells and tumour cells through various pathways, exhibiting both anti‐tumoural and pro‐tumoural functions. They can participate in anti‐tumour responses by directly killing tumour cells or through interactions with other immune components. However, in many human tumours, a high density of TANs is correlated with a poor prognosis, likely due to their role in promoting inflammation that supports tumour growth.

## 2. Neutrophil Production and Its Migration to TME

Neutrophils originate from myeloid precursors in the bone marrow. They make up 10%–25% of circulating leukocytes in mice and 50%–70% in humans [1]. These cells have a short lifespan in peripheral blood, necessitating continuous replenishment from the bone marrow. This replenishment relies on the granulocyte colony‐stimulating factor receptor (G‐CSFR) signalling pathway [2]. Studies have shown that a deficiency in G‐CSFR or G‐CSF results in severe neutropenia in both mice and humans. Maintaining neutrophil homeostasis, particularly during an inflammatory response, also depends on other cytokines, such as interleukin‐6 (IL‐6) and granulocyte–macrophage colony‐stimulating factor (GM‐CSF) [3].

The movement of neutrophils from the bone marrow to the peripheral blood is precisely controlled by chemokines, with CXC‐chemokine receptor 2 (CXCR2) and CXCR4 playing crucial roles. Bone marrow stromal cells continuously produce CXCL12, which binds to CXCR4, providing a retention signal for CXCR4‐positive neutrophil progenitors and immature neutrophils. Conversely, CXCR2 signalling interacts antagonistically with CXCR4. The release of neutrophils from the bone marrow into the blood is controlled by the downregulation of CXCR4 and the subsequent upregulation of the CXCR2 receptor [4, 5].

**Figure 1:**
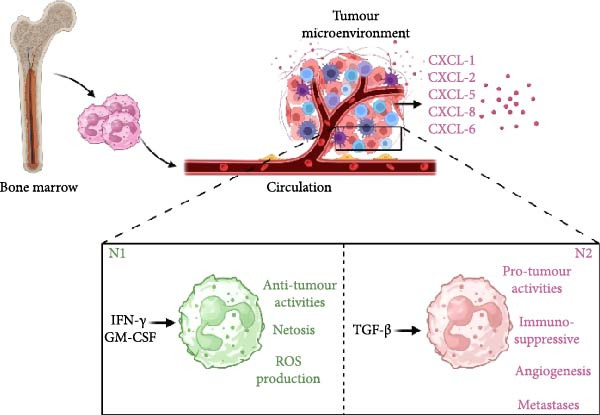
Migration of neutrophils from bone marrow to the tumour microenvironment and neutrophil subtypes in the tumour microenvironment. The production of neutrophil‐attracting chemokines such as CXCL1, CXCL2, CXCL5, CXCL8 and CXCL6 causes neutrophil infiltration into the tumour microenvironment. Neutrophils may be polarised to N1 or N2 subtypes based on the cytokine milieu in the tumour microenvironment. N1 and N2 subtypes exhibit anti‐tumoural and pro‐tumoural activities, respectively. CXCL8 is also known as Interleukin‐8 (IL‐8).

The recruitment of neutrophils towards transformed or cancer cells depends on interleukin‐8 (IL‐8), which attracts CXCR1/CXCR2‐expressing neutrophils to sites of tissue damage and cancer [6, 7]. In the context of neoplasia, the production of growth factors such as GM‐CSF and G‐CSF, along with inflammatory cytokines like IL‐1*β*, IL‐6 and IL‐17, is increased by tumour cells, tumour‐infiltrating leukocytes and tumour‐associated stromal cells. Moreover, tumour cells, as well as cancer‐associated endothelial cells and fibroblasts, express chemokines such as CXCL1, CXCL2, CXCL5, CXCL6 and CXCL8 (IL‐8). These chemokines strongly recruit neutrophils to tumours [8] (Figure 1).

## 3. Neutrophil Plasticity in Tumour

Neutrophils are a significant component of the TME, but their roles remain controversial and context‐dependent. These TANs are present in several types of cancers, including head and neck, hepatocellular carcinoma (HCC), melanoma, glioblastoma, pancreatic ductal carcinoma, colorectal cancer and renal cell carcinoma (RCC). Most studies indicate that increased levels of neutrophils within tumour tissue are associated with poor patient prognosis [9]. However, this evidence does not definitively establish that neutrophils are directly responsible for cancer progression. Heterogenous neutrophil populations displaying different functions and morphologies have been identified within the circulation and tumour tissues of cancer patients. Considering their location and maturation status profile, T1 and T2 subpopulations are mostly at tumour periphery as T2 are more mature than T1. T3 subpopulation are mixed maturity population which are found predominantly in the tumour core and exhibit glycolytic and hypoxic gene signatures [10].

Generally, based on their anti‐tumoural or pro‐tumoural activities, TANs are classified as N1 and N2, respectively. However, neutrophil subpopulations in tumours exhibit a spectrum rather than definite discrete N1 and N2 types. There are intermediate states with overlapping phenotypes and functions influenced by tumour microenvironment signals [11]. In cancer patients, circulating neutrophils also generally can be divided into subpopulations according to their densities when isolated and centrifuged. Normal or high‐density neutrophils (HDNs) are mostly considered anti‐tumour cells, while low‐density neutrophils (LDNs) are thought to promote tumour activity in malignancies and sometimes overlapping phenotypically with granulocytic PMN–MDSCs. Although, NDNs (normal‐density neutrophils) sometimes show immunosuppressive features and can suppress T cell proliferation, differing from NDNs in healthy individuals which are more classically proinflammatory. The LDNs also could be divided into CD45 high LDNs that are mature with immunosuppressive capacity and CD45 low LDNs that are immature with less suppressive activities [12]. These neutrophils can switch between these states in response to different environmental factors [13].

N1 neutrophils differentiate in the presence of Type I interferons, such as interferon‐*β* (IFN*β*). The combination of GM‐CSF and IFN*γ* can also induce N1 differentiation. This subtype is characterised by increased adhesion molecules (ICAM‐1), enhanced transmigration, phagocytosis, oxidative burst (producing reactive oxygen species [ROS]), FAS expression, degranulation and the release of NETs (NETosis) [14, 15]. ROS, such as superoxide anion (O_2_^−^), hydrogen peroxide (H_2_O_2_) and nitric oxide (NO), play a central role in neutrophil‐mediated tumour lysis [16]. Moreover, NET components induce tumour cell cytotoxicity and expelled DNA strands from NETs trap tumour cells, impairing their metastatic and proliferative capacities [17].

TANs express Fc receptors and can kill antibody‐opsonised tumour cells via antibody‐dependent cellular cytotoxicity (ADCC). A cluster of N1 neutrophils is characterised by CD86 and HLA‐DR expression, demonstrating antigen‐presenting capabilities that enhance the anti‐tumour effect of T cells [18]. Conversely, high concentrations of transforming growth factor‐*β* (TGF‐*β*) can polarise neutrophils into the N2 subtype, which exhibits opposing functions to the N1 subtype. N2 neutrophils are characterised by their strong immunosuppressive and tumour‐promoting activities, contributing to tumour angiogenesis, invasion and metastasis through various factors. N2 neutrophils efficiently recruit Tregs into the TME through the chemokine C–C motif ligand 17 (CCL17). Additionally, they produce immune suppressor mediators like ARG1, which inhibit T‐cell effector functions [14, 19].

The primary binary classification of neutrophils into N1 and N2 is an oversimplification, as neutrophils exhibit significant plasticity, particularly in complex conditions like the TME. The association of TANs with survival outcomes in cancer patients remains controversial and has garnered significant attention in recent research. Previous studies have consistently linked high levels of intra‐tumoural neutrophils to poorer survival outcomes across various cancer types [20], likely due to the overall cytokine milieu and metabolic conditions in the TME, which often drive neutrophils to adopt the N2 phenotype (Figure 1).

## 4. NETs and TME

Neutrophils undergo the process of NETosis to trap and kill various bacterial, fungal and protozoal pathogens. Initially, this process was viewed as a beneficial mechanism evolved by the immune system to protect the body. However, research in oncology has revealed that NETosis has multifaceted roles in advancing cancer and contributing to therapy resistance. For example, studies such as those by Liu et al. [21] have demonstrated that BCG‐induced NETosis plays a significant role in bladder cancer treatment by inducing apoptosis, causing cell‐cycle disruption and inhibiting tumour cell migration. Conversely, in other cancers, such as gastric and breast cancer, NETosis promotes metastasis. These contradictory findings have sparked increased research interest in this topic from 2006 to 2024.

**Figure 2:**
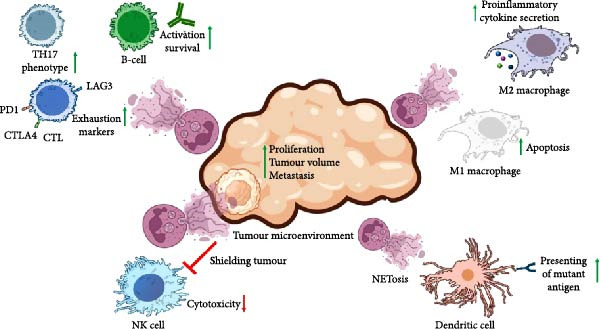
NETosis affects the tumour microenvironment and immunosurveillance. It promotes cancer cell proliferation, increases tumour volume and facilitates metastasis. Additionally, NETosis impairs NK cell cytotoxicity by reducing their migration and infiltration into the tumour microenvironment. NETosis also influences adaptive immunity by driving CD8^+^ T‐cell exhaustion through the upregulation of exhaustion markers and skewing CD4^+^ T‐cell polarisation toward the TH17 phenotype. Its effects on macrophages are phenotype‐dependent; NETosis induces apoptosis in M1 macrophages while enhancing the secretion of pro‐inflammatory cytokines in M2 macrophages. Furthermore, dendritic cells can internalise extracellular antigens released during NETosis, which, in the case of mutations such as those in acute myeloid leukaemia, may enhance antigen presentation and immune activation. NETosis also regulates B‐cell activation and migration.

One possible explanation for the detrimental role of NETosis in cancer is the substantial alterations it induces in the TME. Several studies propose that the TME acts as an inducer of NETosis, initiating a sequence that begins with the TME, continues with NETosis and culminates in tumour growth, invasion, metastasis and angiogenesis, as shown in Table 1. Moreover, NETosis not only influences these processes but also suppresses tumour immunosurveillance (Figure 2).

**Table 1:** Impact of NETosis on tumour microenvironment: interactions with immune cells and tumour progression.

Tumour progression			
Cancer type	Process	Mechanism	Study
Gastric cancer	Metastasis	The co‐culturing of activated neutrophils with cancer cells resulted in the upregulation of E‐cadherin and vimentin expression, leading to increased cancer cell migration. However, no significant alterations were observed in cell proliferation or cell cycle progression.	[22]
Cholangiocarcinoma	Metastasis/angiogenesis	Through CCK8 assay, activated neutrophil which were induced for NETosis formation showed that efficiently strengthened the proliferation and metastasis via ITGAV/NFκB.	[23]
Breast cancer	Metastasis	Lung mesenchymal stromal cells at the pre‐metastatic stage express complement 3 (C3). C3 promotes neutrophil recruitment and the formation of NETosis, thereby facilitating the metastasis of cancer cells to the lungs.	[24]
Gastric cancer	Metastasis	NETosis promote gastric cancer metastasis by increasing NAT10‐mediated N4‐acetylcytidine modification of SMYD2 mRNA, which stabilises SMYD2 and enhances cancer cell invasion and migration.	[25]
Colorectal liver metastases cancer	Proliferation	Neutrophil elastase released from NETosis activates the TLR‐4 signalling pathway in cancer cells. This activation enhances the expression of mitochondrial biogenesis‐related proteins, including PGC‐1*α*, thereby promoting increased energy production and subsequently accelerating tumour cell proliferation.	[26]
Breast cancer	Metastasis	NETosis promote breast cancer progression by activating the tissue factor (TF)/PAR2 signalling pathway, which triggers pro‐inflammatory and pro‐tumour gene expression through MAPK signalling. Blocking TF or PAR2 reduced these effects, suggesting that the TF/PAR2 axis mediates the pro‐tumour impact of NETs on breast cancer cells.	[27]
Lung cancer	Metastasis	Inflammation‐induced NETosis reactivates dormant cancer cells by releasing proteases (neutrophil elastase and MMP9) that remodel laminin and activate integrin signalling, leading to metastasis.	[28]
Lung cancer	Metastasis	NETosis can capture circulating tumour cells, thereby facilitating metastatic dissemination. This entrapment is mediated through interactions involving *β*1‐integrin.	[29]

**Immunosurveillance**			
**Cell**	**Process**	**Mechanism**	

NK cell	↓Cytotoxicity, ↓migration and ↓motility	NETosis provides a protective barrier that shields tumour cells from the cytotoxic activity of natural killer (NK) cells. Furthermore, NET‐derived MMP9 contributes to NK cell dysfunction, thereby facilitating tumour invasion.	[30–32]
CTL	Exhaustion↑	NETosis enhances the expression of exhaustion markers, including PD‐1, Tim‐3 and Lag‐3, on the surface of CD8+ T cells. This upregulation impairs their effector function by altering cytokine production and disrupting cellular metabolism.	[33]
Macrophage	Secretion of proinflammatory cytokine	NETosis enhances inflammation by modulating macrophage function and phenotype diversity. Nakazawa et al. showed that both M1 and M2 macrophages attempt to degrade NETs, but during this process, M1 macrophages undergo apoptosis, whereas M2 macrophages shift from an anti‐inflammatory to a pro‐inflammatory response. Similarly, Zhang et al. showed that NETs increase the release of pro‐inflammatory cytokines from macrophages when co‐cultured with A549 lung cancer cells.	[34, 35]
DC	Displaying leukaemia‐associated antigens	NETosis can improve the effectiveness of dendritic cell (DC) vaccines against acute myeloid leukaemia (AML). NETosis promotes the exposure of mutant nucleophosmin (NPMc+), which relocates from the nucleolus to the cytoplasm when mutated. These antigens can be utilised as a source for DC‐mediated antigen presentation.	[36]
T‐helper	Modulating polarisation	NET can induce TH17 polarisation when it is accompanied with CD3/CD28 stimulation.	[37]
B‐cell	Activating and attracting B‐cells	Sangaletti et al. showed that NETs, through their interaction with CD5^+^ B cells, drive the progression from autoimmunity to lymphoma in a mouse model of B‐cell chronic lymphocytic leukaemia. They also found that B cell–helper neutrophils release higher levels of B cell–activating and attracting factors—BAFF, APRIL, CD40L, IL‐21 and CXCL12—along with increased NET production.	[38, 39]

## 5. Therapeutic Targeting of Neutrophil Plasticity and NETosis

Understanding the impact of inflammatory chemokines and cytokines on neutrophil activity has become a key focus in cancer research, particularly as these cells are considered potential targets for new immunotherapies. Neutrophil heterogeneity in the liver tumour microenvironment has been demonstrated by recent single‐cell investigations. Different subsets of neutrophils have either pro‐tumour or anti‐tumour characteristics; certain populations display immunosuppressive markers and active NETosis [40]. According to a recent study, tumour‐infiltrating neutrophils alter their NETotic activity through functional reprogramming. Specific neutrophil subsets in the TME have been shown to enhance tumour survival and resistance to therapy, suggesting that NETosis serves as a potential therapeutic target as well as a biomarker [41] Furthermore, NETs have been directly related to increased mortality rates and less favourable outcomes in cancer patients. Potential treatment strategies for limiting the spread and metastasis of cancer may involve targeting pathways that prevent the formation of NETs [42]. This section will discuss two emerging and urgent approaches to targeting neutrophils: limiting neutrophil recruitment and polarisation and preventing NET formation.

### 5.1. Inhibition of Neutrophil Recruitment, Polarisation and Function as a Therapy

After infiltrating the TME, TANs can be polarised from an anti‐tumour (N1) phenotype to a pro‐tumour (N2) phenotype [43]. TGF‐*β*, an immunosuppressive cytokine, controls this TAN phenotypic switch in the TME, inhibiting the anti‐tumour activity of T cells and NK cells [44]. It is believed that TAN polarisation to the N2 type can be reversed by blocking the TGF‐*β* pathway, which inhibits colorectal cancer growth [45]. Additionally, inhibiting TGF‐*β* in non‐small cell lung cancer (NSCLC) also halts tumour growth by polarising TANs towards anti‐tumour phenotypes [46]. Another study demonstrated that removing TGF‐*β* from myeloid cells inhibited tumour metastasis. Conversely, reintroducing TGF‐*β*‐producing myeloid cells in tumour‐bearing mice restored the suppressed metastatic phenotype. This effect was mediated by reductions in TGF‐*β*1, Type II cytokines, arginase 1 and iNOS, which increased IFN‐*γ* expression and stimulated systemic immunity [47]. The TGF‐*β* pathway also significantly influences neutrophil recruitment to tumours and subsequent cancer resistance to immune checkpoint inhibitors [42, 48–50]. Clinical trials are currently underway to investigate the effectiveness of combining immune checkpoint inhibitors with galunisertib in the treatment of solid tumours (NCT02734160). Therefore, the role of TGF‐*β* in stimulating systemic immunity suggests that suppressing the TGF‐*β* pathway and immunological checkpoints together may enhance the effectiveness of future immunotherapeutic drugs. Systemic inhibition of the TGF‐*β* pathway has therapeutic potential; however, there are still major challenges. Due to TGF‐*β* has a variety of roles in vascular homeostasis, immunological regulation and tissue regeneration, uncontrolled inhibition of this can result in negative outcomes such inflammation, fibrosis and cardiotoxicity [51]. Additionally, the coexistence of tumour‐promoting and tumour‐suppressive effects makes clinical implementation difficult and emphasises the necessity of combination or context‐specific therapeutic methods [52, 53].

Moreover, CXCL8 (IL‐8) has been identified as a crucial neutrophil attractant and a significant determinant of neutrophil activation. Increasing evidence underscores the significant role of the CXCL8–CXCR1/2 axis in the TME, along with the predictive significance of serum CXCL8 levels in human cancers following immune checkpoint blockade therapy [54–56]. In advanced NSCLC, patients showing a positive response to nivolumab, a PD‐1‐targeted therapy, displayed elevated BMP‐9 levels and reduced levels of CXCL8, TNF‐*α* and IP10 compared to non‐responders [57]. The potential of CXCL8 as a therapeutic target is underscored by the fact that an anti‐CXCL8 monoclonal antibody can enhance antitumour immunity in triple‐negative breast cancer and inhibit the recruitment of neutrophils into the tumour. The huMax‐IL8 monoclonal antibody effectively reduces the infiltration of PMN–MDSCs into the TME and enhances the efficacy of immunotherapy by directly targeting CXCL8 [58].

IL‐17 is crucial to the immune response and tumour progression [59]. Findings suggest that IL‐17 recruits neutrophils to the TME, contributing to breast tumour metastasis and treatment resistance [60]. According to a recent study, IL‐17 increases neutrophil numbers and NET formation in the TME, leading to resistance to immunotherapy and immunosuppression. However, in pancreatic cancer, IL‐17 inhibition increases sensitivity to PD‐1 and CTLA4 offering potential improvements in cancer treatment strategies [61]. Furthermore, activating NK cells has shown that treating pro‐tumour neutrophils with IFN‐*γ* and TNF‐*α* can alter their role from tumour promotion to tumour suppression. This finding suggests that using normal NK cells might offer an effective therapeutic strategy for cancer treatment [62]. In melanoma patients receiving Type I IFN treatment, neutrophil activation shifts to an antitumour state. This treatment results in increased ICAM1 expression, enhanced anti‐tumour activity of neutrophils, increased spontaneous neutrophil death and a prominence of immature neutrophils in the patient’s blood [63].

The immunosuppressive capacity of neutrophils can be reduced by focusing on key pathways that support immunosuppression and regulate their activity [64]. ARG1, primarily triggered by polymorphonuclear granulocytes (PMNs) in humans, is linked to the acceleration of tumour growth and enhanced immunosuppression [65–67]. Additionally, ARG1 serves as a prognostic biomarker in various types of cancer [68–70]. T cell receptor expression and proliferation are suppressed when ARG1 is present in the TME, whereas PMN‐mediated T cell suppression is avoided when ARG1 is inhibited [71]. Several ARG1 inhibitors, such as OATD‐02 and CB‐1158, have recently been selected for clinical trials in tumour immunotherapy [72]. It has been discovered that CB‐1158 efficiently inhibits human ARG1 [73]. Increased ARG1 expression in epithelial ovarian cancer is associated with immune suppression and tumour growth, whereas ARG1 inhibition reduces ARG1‐mediated immune suppression and tumour progression [74]. Additionally, clinical trials are being undertaken to investigate the efficacy of combining immune checkpoint inhibitors and CB‐1158 for the treatment of solid tumours (NCT03314935, NCT02903914 and NCT03361228).

### 5.2. NETs as a Target for Cancer Therapy

An attractive approach for tumour therapy may be to target NETs. Targeting NETs can be accomplished through various strategies, including preventing NET‐tumour contact, disrupting NET structure and blocking pathways involved in NET formation [75]. IL‐1*β* is capable of inducing NETosis, whereas the formation of NETs can cause TGF‐*β*‐dependent EMT and chemotherapy resistance in tumour cells. After chemotherapy, the use of IL‐1*β*‐blocking antibodies has been shown to decrease the number of metastatic foci in the lungs, inhibit NET production and reduce neutrophil accumulation [76]. CXCL8 has been shown to promote the production of NETs [17]. Furthermore, CXCL8 creates a positive feedback loop between colorectal cancer liver metastases and NETs [77]. According to one study, CXCL8 stimulates NET production by interacting with CXCR2, which in turn increases the migration and proliferation of cancer cells [78]. Thus, targeting the CXCL8‐CXCR1/2 axis, which has significant therapeutic potential, may increase the effectiveness of immune checkpoint inhibitors. Kaiser et al. [79] have also recently examined the critical impacts of CXCL8 inhibition on neutrophil activation and NET production. Their findings indicate that in mice, blocking the CXCL8‐CXCR1/2 axis with an anti‐IL‐8 antibody or a commercially available CXCR blocker results in reduced neutrophil activation and NET production (Table 2).

**Table 2:** Therapeutic approaches that target NETs in cancer.

Therapeutic target	Agent	Cancer type	Stage	Mechanism	References
IL‐1*β* inhibition	Anti‐IL‐1*β* antibody	Non‐small cell lung cancer	Preclinical	Reduces NET formation and metastasis; reverses chemotherapy‐induced TGF‐*β* activation	[68]
CXCL8–CXCR1/2 axis blockade	Riparixin (allosteric CXCR1/2 inhibitor)	Metastatic HER2‐negative breast cancer	Clinical–Phase Ib	Paclitaxel; inhibits breast cancer stem cells and NETosis	[73]
	SX‐682 (CXCR1/2 inhibitor)	Head & neck	Preclinical	Reduces CXCR2^+^ MDSC trafficking and enhances NK/T‐cell infiltration, improving anti‐tumour immunotherapy efficacy	[74, 75]
CXCL8–CXCR1/2 axis blockade	Anti‑IL‑8 antibody/CXCR blocker	Inflammatory disease models	Preclinical/translational	Reduction of neutrophil activation and NET formation in vivo	[72]
IL‐17–induced NETosis	IL‐17 blockade	Pancreatic cancer	Preclinical	Blocking IL‐17‐induced NETs enhances response to PD‐1/CTLA‐4 checkpoint blockade	[54]
NET degradation	DNase I (recombinant enzyme) AAV‐DNase I (gene therapy vector)	Breast cancer (lung metastasis), hepatocellular carcinoma (HCC), colorectal cancer (liver metastasis)	Preclinical/translational	Degrades NET DNA; reduces metastasis and tumour inflammation	[76–81]
PAD4 inhibition	GSK484, Cl‐amidine, BMS‐P5 (PAD4 inhibitors)	Breast cancer (pulmonary metastasis), melanoma, multiple myeloma (murine models)	Preclinical	PAD4 inhibition suppresses NET formation, limiting metastasis and tumour progression.	[82–86]
NET–DNA receptor blockade	Antibodies against CCDC25 (NET–DNA receptor)	Breast cancer (MDA‐MB‐231)	Preclinical	Blocks NET‐DNA binding; reduces liver metastasis	[87]
PR3 inhibition	Sivelestat (PR3 inhibitor)	Breast cancer	Preclinical	Inhibits neutrophil recruitment and NETosis, suppressing lung metastasis	[78]

In a mouse pancreatic cancer model, inhibition of IL‐17‐stimulated NETosis has shown potent anti‐cancer effects and enhances sensitivity to ICIs (PD1, CTLA4) [61]. NETosis can be triggered by activating neutrophil surface receptors CXCR1/2. The combination of CXCR1/2 inhibitors and ICI is currently being explored in several clinical trials aimed at reducing NETosis. In a Phase Ib clinical trial involving patients with metastatic HER‐2‐negative breast cancer, riparexin—an allosteric CXCR1/2 inhibitor—demonstrated promising activity against breast cancer stem cells when used in combination with paclitaxel [80]. Additionally, preclinical evaluation is in progress for SX682, an orally available small molecule that acts as an allosteric inhibitor of CXCR1/2 [81]. The mechanism of action of this inhibitor may include increasing the accumulation and activation of KIL cells, reducing the tumour accumulation of CXCR2+ MDSCs in mice, or improving the efficacy of T‐cell immunotherapy [81, 82] (Table 2).

DNase I, an endonuclease that degrades NET DNA, is FDA‐approved for cystic fibrosis [83, 84]. DNase I treatment has been shown to reduce lung metastases and breast cancer spheroid growth [30, 85]. NETs induce a tumour inflammatory response that facilitates the spread of HCC. In a mouse model of HCC, the anti‐inflammatory drugs aspirin and hydroxychloroquine, when combined with DNase I, successfully reduced intrahepatic and pulmonary metastases [86]. The AAV DNase I, a viral gene therapy vector linked to DNase I expression, blocked the growth of liver metastases from colorectal cancer by lowering neutrophil recruitment and preventing NET formation in human tumour cells [87].

Peptidyl arginine deiminase 4 (PAD4) is a crucial enzyme involved in the formation of NETs. Suppressing PAD4 has been shown to mitigate the pro‐tumour effects of NETs in various disease models [88, 89]. Several substances have been documented to inhibit the function of PADs, such as BMS‐P5 and Cl‐amidine. These are novel inhibitors that effectively prevent the formation of NETs and reverse disease progression [90, 91]. The A2A receptor (CGS21680) and another novel PAD4 inhibitor (GSK484) effectively prevent NET formation [92]. GSK484 prevents NETosis and pulmonary metastasis induced by CC overexpression of AT3 breast cancer cells [85]. Cl‐amidine inhibits melanoma growth like GSK484 [93]. The new inhibitor BMS‐P5 can prevent the formation of NETs, thereby potentially delaying the progression of multiple myeloma [94]. When GSK484 or Cl‐amidine is used to inhibit NET production, tumour colonisation is reduced [93]. In conclusion, these findings suggest that targeting PAD4 in cancer may have therapeutic benefits (Table 2).

**Figure 3:**
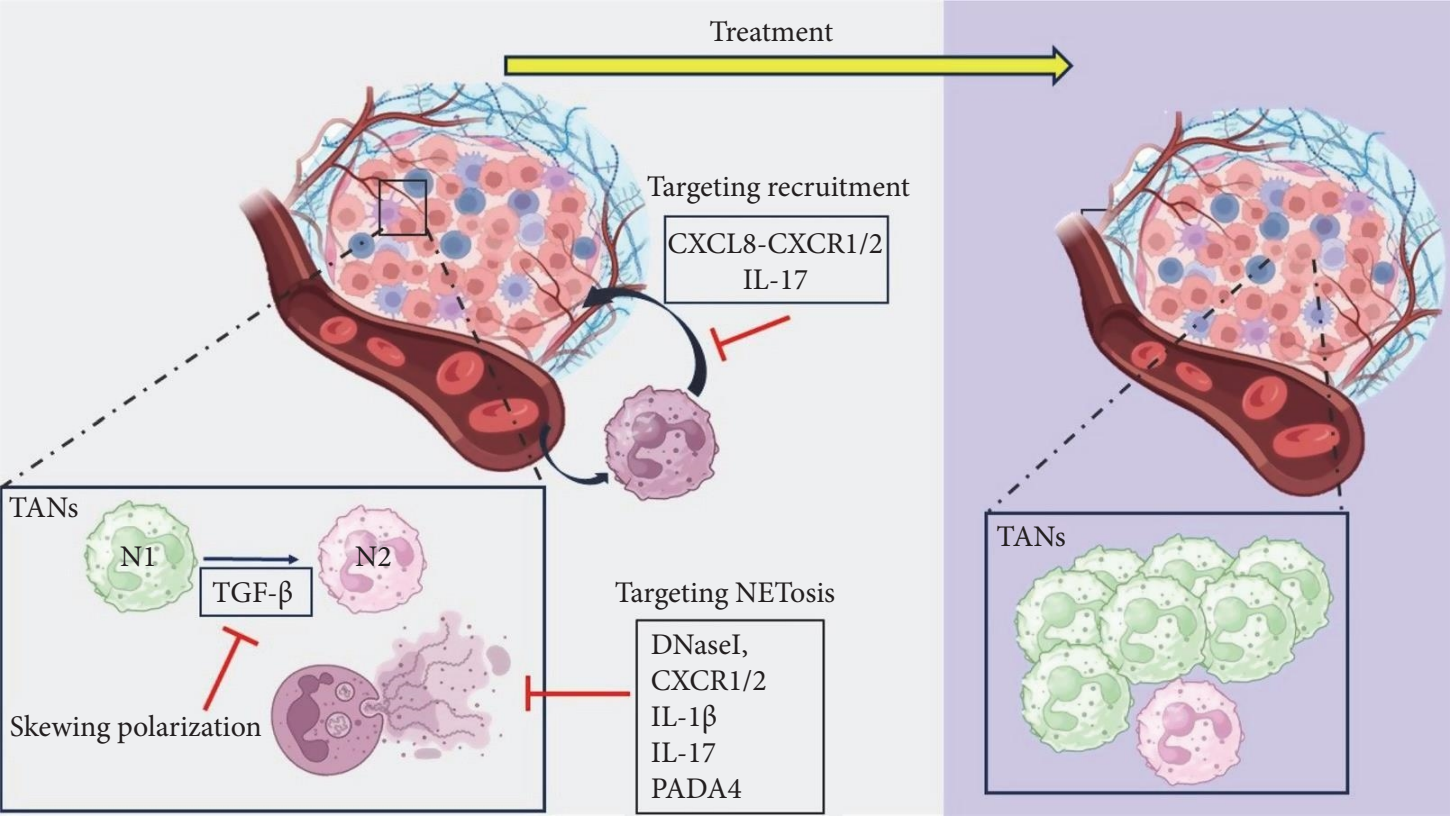
Therapeutic strategies for targeting neutrophils in cancer. DNase I, CXCR1/2, IL‐1*β*, IL‐17 and PAD4 could be potential therapeutic targets for inhibiting NETosis and controlling cancer progression and metastasis. Blocking the TGF‐*β* pathway reverses tumour‐associated neutrophil (TAN) polarisation to the N2 type. Targeting the CXCL8‐CXCR1/2 axis and IL‐17 reduces neutrophil recruitment to the tumour microenvironment. NETs: Neutrophil extracellular traps; tumour microenvironment; PAD4: peptidylarginine deiminase 4; CXCR1/2: C‐X‐C chemokine receptor 1 and 2; IL‐1*β*: interleukin‐1*β*; IL‐17: interleukin‐17; transforming growth factor *β* (TGF‐*β*); CXCL8 is also known as interleukin‐8 (IL‐8). The following interventions are displayed based on the developmental stage: clinical (TGF‐*β* inhibition), preclinical to early clinical (CXCR1/2 inhibition) and preclinical (IL‐17, PAD4, DNase I, CCDC25, PR3 targeting).

Polyclonal antibodies that block CCDC25, the NET‐DNA receptor, effectively inhibited NET‐induced cell migration in vitro and reduced liver metastasis of MDA‐MB‐231 cells in NOD/SCID mice in vivo [95]. A PR3 inhibitor called Sivelestat successfully reduced CC‐induced neutrophil recruitment and NETosis [85]. These findings highlight the collaborative role of tumour cells, TANs and NET formation in the TME, as well as the importance of NETs in tumour metastasis. They also raise the possibility of combining immunotherapy with NET inhibition in clinical practice (Table 2) (Figure 3).

## 6. Summary and Conclusion

Neutrophils are pivotal components of the TME, with their functional impact determined by their polarisation into pro‐tumoural or anti‐tumoural phenotypes. The recruitment of TANs by tumours extends their lifespan compared to circulating neutrophils and alters their phenotype and functions, thereby promoting tumour development. Currently, neutrophils and their derivatives are being investigated as targets for the early detection and treatment of cancer, though these therapeutic approaches remain in the early stages of development. NETosis also significantly impacts cancer cells and immune components within the TME. The therapeutic potential of targeting neutrophils and NETosis to prevent metastasis, reverse therapy resistance and enhance immune responses offers new pathways in cancer treatment. Despite their potential, these therapies remain in the early stages of development. Future research should focus on identifying biomarkers for NET‐related therapy resistance, conducting longitudinal studies to monitor changes in neutrophils and NETs over time and optimising NET‐targeting treatments for clinical application.

## Consent

The authors have nothing to report.

## Disclosure

All authors reviewed and revised the text. All authors read and approved the final version of the work to be published.

## Conflicts of Interest

The authors declare no conflicts of interest.

## Author Contributions

All authors contributed equally to the conception and the main idea of the work. Mojdeh Soltani, Sara Falahi and Mohammad Abbaszadeh draughted the main text, figures and tables. Nahid Eskandari supervised the work and provided comments and additional scientific information. Mark J. M. Sullman and Hamed Fouladseresht reviewed and revised the text. All authors contributed to responding to the reviewer’s comments.

## Funding

The authors received no specific funding for this work.

## Data Availability

All data supporting the findings of this study are included within the article.
